# Novel Approaches Outside the Setting of Immunotherapy for the Treatment of Multiple Myeloma: The Case of Melflufen, Venetoclax, and Selinexor

**DOI:** 10.3389/fonc.2021.716751

**Published:** 2021-09-30

**Authors:** Nicola Sgherza, Paola Curci, Rita Rizzi, Pellegrino Musto

**Affiliations:** ^1^ Unit of Hematology and Stem Cell Transplantation, Azienda Ospedaliero Universitaria Consorziale (AOUC) Policlinico, Bari, Italy; ^2^ Department of Emergency and Organ Transplantation, “Aldo Moro” University School of Medicine, Bari, Italy

**Keywords:** multiple myeloma, relapsed/refractory disease, melflufen, venetoclax, selinexor

## Abstract

Although the survival rate of patients with multiple myeloma has significantly improved in the last years thanks to the introduction of various classes of new drugs, such as proteasome inhibitors, immunomodulatory agents, and monoclonal antibodies, the vast majority of these subjects relapse with a more aggressive disease due to the acquisition of further genetic alterations that may cause resistance to current salvage therapies. The treatment of these often “triple” (or even more) refractory patients remains challenging, and alternative approaches are required to overcome the onset of that resistance. Immunotherapies with novel monoclonal, drug-conjugated, or bi-specific antibodies, as well as the use of chimeric antigen receptor T cells, have been recently developed and are currently investigated. However, other non-immunologic therapeutic regimens based on melfluflen, venetoclax, or selinexor, three molecules with new mechanisms of action, have also shown promising results in the setting of relapsed/refractory myeloma. Here we report the most recent literature data regarding these three drugs, focusing on their efficacy and safety in multiple myeloma.

## Introduction

Multiple myeloma (MM) is the second most common hematological cancer (1). Despite the survival of patients affected by this plasma cell neoplasm has improved over the past years thanks to the advent of very effective drugs, such as proteasome inhibitors (PIs), immunomodulatory agents (IMiDs), and monoclonal antibodies (MoAbs), most of these subjects usually experience an alternation of remission and relapse (2, 3) as they cycle through therapeutic options. Typically, each remission is usually shorter than the last as the tumor becomes more aggressive, with progression and treatment resistance driven by clonal evolution and genomic instability within myeloma clones (4, 5). Moreover, since MM patients are usually elderly, they often present with comorbidities, such as disabilities, diabetes, and pulmonary and cardiovascular diseases, which not only further impact the quality of life of the patient but also limit the therapy options (6, 7). Treatments for relapse largely depend on prior therapy, according to previous response and tolerability, with class switching often prioritized (8). Many new approaches that aim to overcome or bypass resistance mechanisms are currently under investigation for patients with relapsed and/or refractory MM (RRMM). Among these, the development of novel monoclonal, drug-conjugated, or bi-specific antibodies (9), as well as the use of chimeric antigen receptor (CAR) T cells (10), have recently opened a new immune-therapeutic scenario for MM patients, ideally integrating or even substituting other “conventional” chemotherapy or PIs/IMiDs-based treatments characterized by a well-known toxicity profile mainly resulting in cytopenia, neurologic symptoms, and thrombophilia. On the other hand, novel, non-immunologic therapeutic regimens based on melfluflen, venetoclax, or selinexor, three molecules with different mechanisms of action, have also shown promising results in the setting of RRMM. These drugs may have the possible advantage of avoiding some specific side effects related to immunological approaches (*i*.*e*., cytokine release syndrome, infusion-related reactions, central nervous system complications, or unusual infections), thus warranting evaluation as possible alternative options or, even better, as partners for new combinations. In this review, we provide an overview of the efficacy and safety, from main clinical trials and real-world experiences, of melfuflen, venetoclax, and selinexor in the setting of RRMM.

## Melflufen

**Figure 1:**
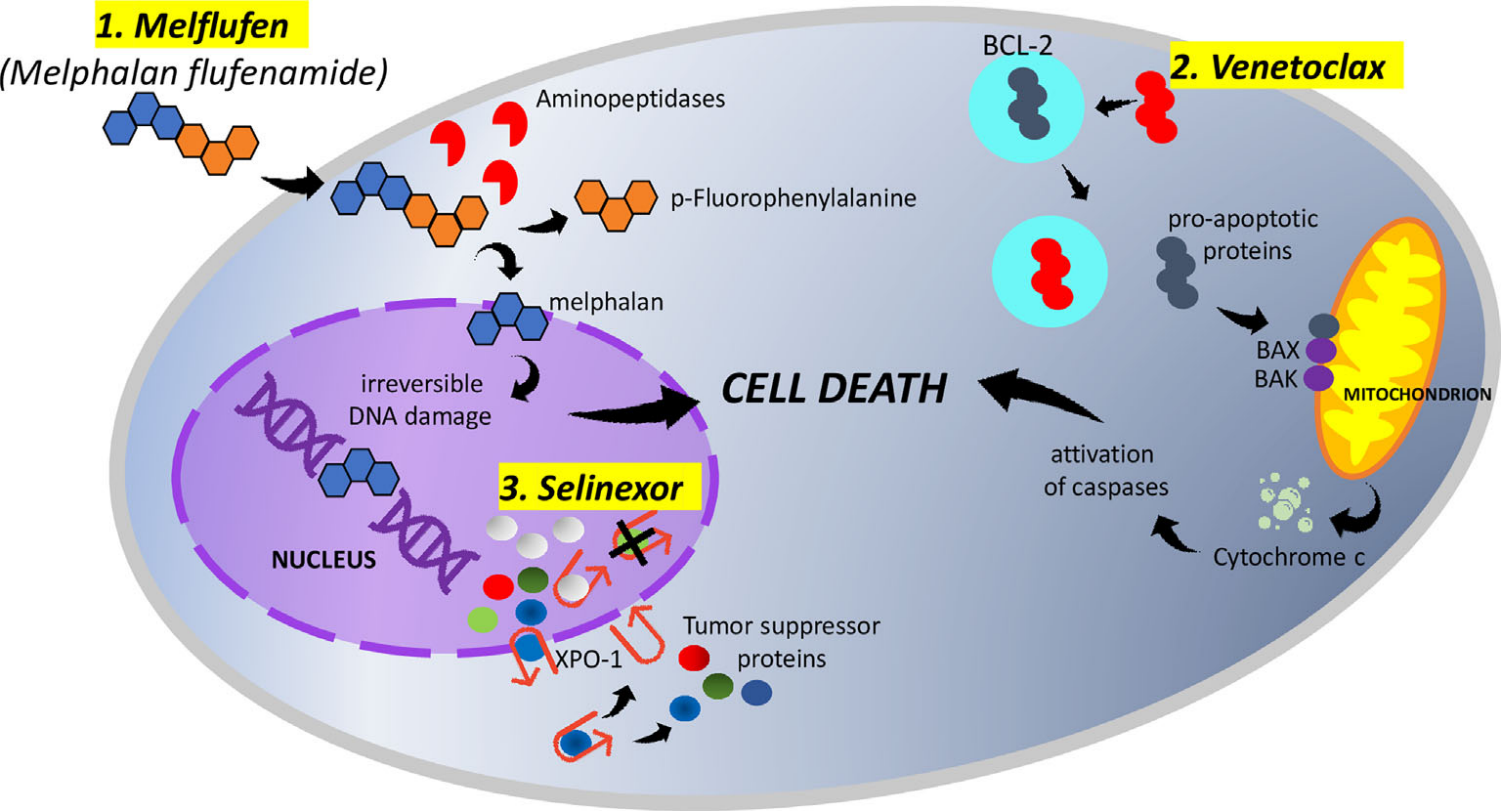
1. Melphalan flufenamide (melflufen) is highly lipophilic and rapidly diffuses across the membranes of myeloma cells. Once inside the cell, aminopeptidases cleave melflufen into melphalan and p-fluorophenylalanine. melphalan accumulates in myeloma cells and, within the nucleus, induces irreversible DNA damage and apoptosis. 2. Venetoclax binds selectively to BCL-2, freeing pro-apoptotic proteins. The released pro-apoptotic proteins associate with the apoptotic effectors BAX and BAK and induce the permeabilization of the mitochondrial outer membrane. The cytochrome c released activates caspases and triggers cell death. 3. Myeloma cells overexpress XPO-1, causing the increased export of tumor-suppressor proteins from the nucleus. Selinexor (represented by white spheres), binding to XPO-1, inhibits the nuclear export of tumor-suppressor proteins (represented by green, blue, and red spheres). The accumulation of tumor suppressors in the nucleus ultimately leads to cell cycle arrest and apoptosis of multiple myeloma cells.

Melflufen (melphalan flufenamide) is a first-in-class peptide–drug conjugate that, through the hydrolytic activity of intracellular aminopeptidases, releases alkylating agents into tumor cells (11, 12). Melflufen is rapidly taken up by myeloma cells due to its high lipophilicity; once inside the cell, aminopeptidases cleave melflufen into melphalan and p-fluorophenylalanine; melphalan accumulates in myeloma cells and, within the nucleus, induces irreversible DNA damage and apoptosis (**Figure 1**) (12–14). Melflufen increases p53 levels, but its cytotoxic activity is not dependent on the activation of p53 function, unlike melphalan; this justifies the activity of melfuflen in melphalan-resistant cells. Moreover, since p53 mutations/deletions can be present at the presentation (10–15%) or at the progression of a disease, a therapeutic approach including melflufen could be considered even in MM patients carrying these genetic alterations (11). Melflufen has also demonstrated an anti-angiogenic activity in *in vitro* and *in vivo* models, inhibitory action on myeloma cell migration, and capacity to overcome the cytoprotective effects of the bone marrow microenvironment. Finally, the combination of melflufen with bortezomib or dexamethasone or lenalidomide triggered a synergistic anti-MM activity *in vitro* (11, 15–17). Preclinical studies provided the framework for different clinical trials. A detailed summary of main clinical trials on melflufen monotherapy or in combination in the setting of RRMM, including schedules and doses, can be found in **Table 1**.

**Table 1:** Summary of findings of main clinical trials with melflufen in relapsed/refractory multiple myeloma.

	Phase/number of patients	Dosing	Median number of prior lines (range)	Efficacy	Adverse events (grades 3 and 4)	Reference
Melflufen +/-Dexamethasone (O-12-M1; **NCT01897714**)	I/23 II/58	**Phase I: M** (15 or 25 or 40 or 55 mg IV) on day 1 of each 21-day cycle; **Dexamethasone** (40 mg) on days 1, 8, and 15 of each 21-day cycle **Phase II:** **a) M** (40 mg IV) on day 1 of each 21- or 28-day cycle; **Dexamethasone** (40 mg) on days 1, 8, and 15 of each 21-day cycle (for any pts on the 28-day treatment schedule, an additional dose of 40 mg dexamethasone was administered on day 22 of each cycle) (**45 pts**) **b) M** (40 mg IV) on day 1 of each 28-day cycle (**13 pts**)	IIa: 4 (3–5) IIb: 5 (4–6)	**IIa** ORR: 31% CBR: 49% VGPR: 11% PR: 20% mPFS: 5.7 months mOS: 20.7 months **IIb:** ORR: 8% CBR: 23% PR: 8% mPFS: 4.4 months mOS: 15.5 months	**IIa:** thrombocytopenia (62%), neutropenia (58%) **IIb:** neutropenia (69%), thrombocytopenia (62%)	Richardson PG et al. (18)
Melflufen plus Dexamethasone (HORIZON, OP-106; **NCT02963493**)	II/157	**M** (40 mg IV): day 1 of each 28-day cycle; **Dexamethasone** (40 mg or reduced dose for patients 75 years or older) on days 1, 8, 15, and 22 of each 28-day cycle	5 (2–12)	ORR: 29% mDOR: 5.5 months mPFS: 4.2 months mOS: 11.6 months	Neutropenia (79%), thrombocytopenia (76%), anemia (43%), pneumonia (10%)	Richardson PG et al. (19)
Melflufen plus Dexamethasone and Daratumumab or Bortezomib (ANCHOR, OP-104; **NCT03481556**)	I-II/ 46	**Daratumumab arm (33 pts):** **M** (30, 40, or 20 mg IV) on day 1 of each 28-day cycle; **Daratumumab** (16 mg/kg) weekly for 8 doses, every other week for 8 doses, and then once every 4 weeks until PD; **Dexamethasone** (20 mg pre-daratumumab and 20 mg/day after-daratumumab; 20 mg total for pts 75 years or older) **Bortezomib arm (13 pts):** **M** (30, 40, or 20 mg IV) on day 1 of each 28-day cycle; **Bortezomib** (1.3 mg/m²) on days 1, 4, 8, and 11; **Dexamethasone** (20 or 12 mg for pts 75 years or older) on days 1, 4, 8, and 11; 40 or 20 mg for pts 75 years or older on days 15 and 22 of each 28-day cycle	2 (1–4) M (30 mg): 3.5 (2–4) M (40 mg): 2 (1–4)	ORR: 70%, mDOR: 12.5 months mPFS: 11.5 months M (30 mg): ORR: 50% M (40 mg): ORR: 71%	Neutropenia (58%), thrombocytopenia (55%), anemia (24%) M (30 mg): thrombocytopenia (50%), neutropenia (33%) M (40 mg): thrombocytopenia (100%), neutropenia (71%)	Ocio EM et al. (20) Hajek R. et al. (21)
Melflufen plus Dexamethasone *versus* Pomalidomide plus Dexamethasone (OCEAN, OP-103; **NCT03151811**)	III/ongoing	**Arm A: M** (40 mg IV) on day 1; **Dexamethasone** (40 or 20 mg for pts 75 years or older) on days 1, 8, 15, and 22 of each 28-day cycle. **Arm B: Pomalidomide** (4 mg orally, daily) on days 1 to 21; **Dexamethasone** (40 or 20 mg for pts 75 years or older) on days 1, 8, 15, and 22 of each 28-day cycle	NA	NA	NA	Schjesvold F. et al. (22)
Melflufen plus Dexamethasone (BRIDGE, OP-107; **NCT03639610**)	II/31	**Arm 1A: M** (40 mg IV) on day 1 of each 28-day cycle; **Dexamethasone** (40 or 20 mg for pts 75 years or older) on days 1, 8, 15, and 22 of each 28-day cycle **Arm 1B: M** (30 mg IV) on day 1 of each 28-day cycle; **Dexamethasone** (40 or 20 mg for pts 75 years or older) on days 1, 8, 15, and 22 of each 28-day cycle **Arm 2A: M** (20 mg IV) on day 1 of each 28-day cycle; **Dexamethasone** (40 or 20 mg for pts 75 years or older) on days 1, 8, 15, and 22 of each 28-day cycle **Arm 2b: M** (30 mg IV) on day 1 of each 28-day cycle; **Dexamethasone** (40 or 20 mg for pts 75 years or older) on days 1, 8, 15, and 22 of each 28-day cycle	NA NA	ORR: 48% CBR: 58% NA	Thrombocytopenia (58%), neutropenia (42%), anemia (35%) NA	Pour L et al. (23)

pts, patients; M, melflufen; ORR, overall response rate; VGPR, very good partial response; PR, partial response; PD, progressive disease; CBR, clinical benefit rate; mPFS, median progression free-survival; mOS, median overall survival; mTTP, median time to progression; mDOR, median duration of response; NR, not reached; NA, not available; IV, intravenous.

O-12-M1 (NCT01897714) is the first study evaluating melflufen in RRMM patients. It is a phase 1/2, multicenter, dose escalation, and dose expansion clinical trial of melflufen +/- dexamethasone in patients who had received two or more prior lines of therapy, including lenalidomide and bortezomib, and were refractory to the last line of therapy (18). In phase 1, among the four doses evaluated (15, 25, 40, and 55 mg), the established melflufen maximum tolerated dose (MTD) was 40 mg; in phase 2, 13 patients received single-agent melflufen and 45 received melflufen plus dexamethasone. With a median follow-up of 28 months, among the 45 patients receiving melflufen plus dexamethasone, the overall response rate (ORR) was 31% (very good partial response, VGPR: five patients; partial response, PR: nine patients), the median progression free-survival (mPFS) was 5.7 months, and the median overall survival (mOS) was 20.7 months. Among the 13 patients who received single-agent melflufen, the ORR was 8%, the mPFS was 4.4 months, and the mOS was 15.5 months. At the last update, with a median follow-up of 46 months, in the arm melflufen plus dexamethasone, mOS and mPFS were unchanged at 20.7 and 5.7 months, respectively (24).

HORIZON (OP-106; NCT02963493) is a pivotal, single-arm, multicenter, phase 2 study evaluating the efficacy and safety of melflufen and dexamethasone in heavily pretreated and poor-risk patients with RRMM refractory to pomalidomide or an anti-CD38 MoAb or both (19). Among 157 efficacy-evaluable patients, ORR was 29%, the median duration of response (mDOR) was 5.5 months, the mPFS was 4.2 months, and the mOS was 11.6 months at a median follow-up of 14 months.

ANCHOR (OP-104; NCT03481556) is a phase 1/2 study evaluating the safety and efficacy of melflufen and dexamethasone in combination with daratumumab or bortezomib in patients with RRMM. In the daratumumab arm, the patients could not have received prior anti-CD38 MoAb therapy; in the bortezomib arm, the patients could not have been PI-refractory. The patients are treated until progressive disease (PD) or unacceptable toxicity. In the daratumumab arm (20), with a median treatment duration of 8.4 months (1.0–23.7), ORR was 70%, including one stringent complete response (sCR), one CR, 10 VGPRs, and 11 PRs. At a median follow-up of 11.9 months, mPFS was 11.5 months and mDOR was 12.5 months. In the bortezomib arm (21), with a median treatment duration of 6.5 months (range: 1.4–29) and 8.7 months (range: 2.1–19.6), ORR was 50%, and it was 71% for melflufen 30 and 40 mg, respectively.

The ongoing, randomized, open-label, phase 3 multicenter study OCEAN (OP-103; NCT03151811) (22) will enroll patients with RRMM following two to four lines of prior therapy and who are refractory to lenalidomide in the last line of therapy. The patients will be randomized to either one of two arms: melflufen plus dexamethasone *versus* pomalidomide plus dexamethasone. The patients will be treated until confirmed PD, unacceptable toxicity, or when the patient or investigator decides to discontinue the therapy.

BRIDGE (OP-107; NCT03639610) is a phase 2 study evaluating the pharmacokinetics of melphalan during treatment with melflufen and dexamethasone in patients with RRMM, following two to four prior lines of therapy and a renal function (creatinine clearance by Cockcroft–Gault formula) between ≥30 to <45 ml/min in cohort 1 and ≥15 to <30 ml/min in cohort 2. The preliminary results on 31 patients have been reported at the 2021 EHA congress with encouraging results; ORR was 48%, and the clinical benefit rate was 58%, with stable renal function (23).

To date, there is no data (or active clinical trials) evaluating the role of melflufen in newly diagnosed MM (NDMM) as well as on any potential impact on stem cells and stem cell collection.

## Venetoclax

The discovery that an increased expression of the oncogene *BCL-2*, located on chromosome 11, prevents cell death and that it is an important factor in tumor survival through the regulation of apoptosis subsequently led to the hypothesis of this pathway as a target for anti-cancer activity (25). Venetoclax (ABT-199), a potent selective inhibitor of the BCL-2 protein, has previously shown an antitumor activity in acute myeloid leukemia (26), non-Hodgkin lymphoma (27), and chronic lymphatic leukemia (28, 29), receiving following approval from FDA and EMA for sub-categories of patients affected by these hematological malignancies. Focusing the attention on the mechanism of action, venetoclax binds selectively to BCL-2, freeing the pro-apoptotic proteins. These molecules associate with the apoptotic effectors BAX and BAK and induce the permeabilization of the mitochondrial outer membrane. Finally, the released cytochrome c activates caspases and triggers cell death (**Figure 1**). Since about 20% of MM patients demonstrate a t(11;14) (that activates BCL-2) and an overexpression of BCL-2, a possible anti-myeloma activity of venetoclax in MM has been investigated. Preclinical studies demonstrated the sensitivity to venetoclax mainly, but not exclusively, in *in vitro* MM cells harboring t(11;14) (30, 31). Moreover, the sensitivity of MM cells to venetoclax would be improved by the addition of dexamethasone (32); venetoclax would enhance bortezomib activity as well. A detailed summary of the main clinical trials on venetoclax monotherapy or in combination in the setting of RRMM, including schedules and doses, can be found in **Table 2**.

**Table 2:** Summary of findings of main clinical trials with venetoclax in relapsed/refractory multiple myeloma (RRMM).

Regimen (trial ID)	Phase/number of patients	Dosing	Median number of prior lines (range)	Efficacy	Adverse events (grades 3 to 4)	Reference
Venetoclax Monotherapy (**NCT01794520**)	I/66	**Venetoclax:** dose escalation cohort (30 pts): 300 to 1,200 mg daily until progression **Venetoclax:** safety expansion cohort (36 pts): 1,200 mg daily until progression	5 (1–15)	**Pts (30): with t(11;14)** ORR: 40%, > VGPR: 27% mTTP: 6.6 months (3.9–10.2) mDOR: 9.7 months **Pts (33) without t(11;14):** ORR: 6%, > VGPR: 6% mTTP: 1.9 months (1.2–2.3)	Thrombocytopenia (26%), neutropenia (21%), anemia (14%), and leukopenia (14%)	Kumar S et al. (33)
Venetoclax plus Dexamethasone (**NCT01794520**)	I/20 II/31	**Venetoclax:** 800 mg daily; **Dexamethasone** 40 mg oral (20 mg for pts ≥75 years of age) on days 1, 8, and 15 of each 21-day cycle	3 (1–7)/ 5 (2–12)	ORR: 60%/48% mTTP: 12.4 months/estimated mTTP: 10.8 months/ mDOR: 12.4 months/estimated DOR at 12 months: 61%	Lymphopenia (20%), thrombocytopenia (10%), neutropenia (10%), anemia (12%), and hypophosphatemia (10%)	Kaufman JL et al. (34)
Venetoclax plus Bortezomib and Dexamethasone (**NCT01794507**)	I/66	**Venetoclax:** dose escalation cohort (54 pts): 100–1,200 mg daily until progression; safety expansion cohort (12 pts): 800 mg daily until progression; **Bortezomib** (1.3 mg/m^2^) on days 1, 4, 8, and 11 during cycles 1 to 8 and days 1, 8, 15, and 22 during cycles 9 to 11); **Dexamethasone** (20 mg on days 1, 2, 4, 5, 8, 9, 11, and 12 during cycles 1 to 8 and on days 1, 8, 15, and 22 during cycles 9 to 11	3 (1–13)	ORR: 67% > VGPR: 42% mTTP: 9.5 months mDOR: 9.7months	Thrombocytopenia (29%) anemia (15%)	Moreau et al. (35)
Venetoclax or Placebo plus Bortezomib and Dexamethasone (BELLINI, **NCT02755597**)	III/291	**Venetoclax** (800 mg daily) (194 pts) or **Placebo** (97 pts); **Bortezomib** (1.3 mg/m²) on days 1, 4, 8, and 11 during cycles 1 to 8 and days 1, 8, 15, and 22 during cycles 9 and beyond; **Dexamethasone** (20 mg) on days 1, 2, 4, 5, 8, 9, 11, and 12 during cycles 1 to 8 and on days 1,2, 8, 9, 15, 16, 22, and 23 during cycles 9 and beyond. Treatment was given in 21-day cycles for the first eight cycles and 35-day cycles from the ninth cycle until PD, unacceptable toxicity, or patient withdrawal	2 (1–3)	mPFS (venetoclax): 23.2 months; mPFS (placebo): 11.4 months mOS (venetoclax): 33.5 months; mPFS (placebo): NR	Neutropenia (21%/8%), thrombocytopenia (15%/30%), anemia (16%/15%), diarrhea (15%/12%), and pneumonia (18%/13%)	Kumar SK et al. (36, 37)
Venetoclax plus Carfilzomib and Dexamethasone (**NCT02899052**)	II/43	**Cohort 1: Venetoclax** (400 mg daily), **Carfilzomib** (27 mg/m^2^) on days 1, 2, 8, 9, 15, and 16; **Dexamethasone** (40 mg) on days 1, 8, 15, and 22 **Cohort 2: Venetoclax** (800 mg daily), **Carfilzomib** (27 mg/m^2^) on days 1, 2, 8, 9, 15, and 16; **Dexamethasone** (40 mg) on days 1, 8, 15, and 22 **Cohort 3: Venetoclax** (800 mg daily), **Carfilzomib** (70 mg/m^2^) on days 1, 8, and 15; **Dexamethasone** (40 mg) on days 1, 8, 15, and 22 **Cohort 4: Venetoclax** (800 mg daily), **Carfilzomib** (56 mg/m^2^) on days 1, 2, 8, 9, 15, and 16; **Dexamethasone** (40 mg) on days 1, 2, 8, 9, 15, 16, 22, and 23	2 (1–3)	ORR: 79%, ≥CR rate: 40% ≥VGPR rate: 64%	Lymphopenia (23%), pneumonia (16%), hypertension (16%), and hypophosphatemia (12%)	Costa L et al. (38, 39)
Venetoclax–Dexamethasone *vs*. Pomalidomide–Dexamethasone in t(11;14)−positive RRMM (CANOVA, **NCT03539744**)	III/ongoing	**Venetoclax** (800 mg daily) or **Pomalidomide** (4 mg daily on days 1–21 of 28-day cycles); **Dexamethasone** (40 mg; 20 mg for patients ≥75 years once weekly)	NA	NA	NA	Mateos M et al. (40)
Venetoclax plus Pomalidomide and Dexamethasone (**NCT03567616**)	II/ongoing	**Part 1:** dose escalation; **Part 2:** dose expansion. For part 2, the participants will be divided into two cohorts based on the presence of t(11;14).	NA	NA	NA	https://clinicaltrials.gov/ (41)
Venetoclax plus Ixazomib and Dexamethasone (**NCT03399539**)	I/II ongoing	**Phase 1: **determine the MTD of **Venetoclax** in combination with **Ixazomib** and **Dexamethasone**; **Phase 2: **evaluate the therapeutic activity of this triplet in patients with relapsed MM	NA	NA	NA	https://clinicaltrials.gov/ (41)
Venetoclax plus Daratumumab and Dexamethasone (VenDd), +/- Bortezomib (V) (**NCT03314181**)	I/II ongoing	**Part 1a: Venetoclax **(various doses administered once daily), **Daratumumab** (1,800 mg SC (preferred) or 16 mg/kg IV; **Dexamethasone** (40 or 20 mg weekly, if necessary, as described in the protocol) **Part 1b: Venetoclax **(at a dose determined by the dose escalation phase), **Daratumumab** [1,800 mg SC (preferred) or 16 mg/kg IV], **Dexamethasone** (40 or 20 mg weekly, if necessary, as described in the protocol) **Part 2a: Venetoclax **(at various doses administered once daily), **Daratumumab** [1,800 mg SC (preferred) or 16 mg/kg IV], **Bortezomib** (1.3 mg/m^2^) cycles 1–8, days 1, 4, 8, and 11; **Dexamethasone** (20 mg) cycles 1–3: days 1, 2, 4, 5, 8, 9, 11,12, and 15; cycles 4–8: days 1, 2, 4, 5, 8, 9, 11, and 12; cycle 9+ weekly (40 or 20 mg weekly, if necessary as described in the protocol) **Part 2b: Venetoclax **(at a dose determined by the dose escalation phase), **Daratumumab** [1,800 mg SC (preferred) or 16 mg/kg IV], **Bortezomib** (1.3 mg/m^2^) cycles 1–8, days 1, 4, 8, and 11; **Dexamethasone** (20 mg) cycles 1–3, days 1, 2, 4, 5, 8, 9, 11, 12, and 15; cycles 4–8: days 1, 2, 4, 5, 8, 9, 11, and 12; cycle 9+ weekly (40 or 20 mg, if necessary as described in the protocol) **Part 3: Venetoclax **(400 or 800 mg once daily), **Daratumumab** [1,800 mg SC (preferred) or 16 mg/kg IV], **Dexamethasone** (40 or 20 mg weekly, if necessary, as described in the protocol) *versus* **Daratumumab** [1,800 mg SC (preferred) or 16 mg/kg IV], **Bortezomib** (1.3 mg/m^2^) cycles 1–8: days 1, 4, 8, and 11; **Dexamethasone** cycles 1–3 (20 mg), days 1, 2, 4, 5, 8, 9, 11,12, and 15; cycles 4–8 (40 or 20 mg weekly, if necessary as described in the protocol): days 1, 2, 4, 5, 8, 9, 11, and 12; cycle 9+ (20 mg) monthly day 1	NA	ORR (VenDd/VenDVd): 96%/92% ≥VGPR rate (VenDd/VenDVd): 96%/79% mPFS and mDOR: NR	**VenDd: **neutropenia (17%), hypertension (12%), fatigue (8%), hyperglycemia (8%) **VenDVd: **insomnia (21%), diarrhea (8%), thrombocytopenia (8%)	Kaufman JL et al. (42)
Cobimetinib +/- Venetoclax +/- Atezolizumab (**NCT03312530**)	Ib/II 49 (ongoing)	**Arm A: Cobimetinib **(60 mg, daily) on days 1–21 of each 28-day cycle until disease progression. **Arm B: Cobimetinib **(40 mg, daily) on days 1–21 of each 28-day cycle; **Venetoclax **(800 mg, daily) on days 1–28 of each 28-day cycle Arm C: **Cobimetinib** (40 mg, daily) on days 1–21 of each 28-day cycle; **Venetoclax **(800 mg daily) on days 1–28 of each 28-day cycle; **Atezolizumab** (at a fixed dose of 840 mg IV) on days 1 and 15 of each 28-day cycle	4 (3–5)	OS (A/B/C): 12.9/12.4/23.3 months ORR (A/B/C):0%/27%/29%	Arms A/B/C: neutropenia (0/14%/38%), anemia (0/23%/24%), thrombocytopenia (0/18%/24%), pneumonia (0/14%/14%)	Schjesvold F. et al. (43)

pts, patients; ORR, overall response rate; VGPR, very good partial response; PR, partial response; PD, progressive disease; CBR, clinical benefit rate; mPFS, median progression free-survival; mOS, median overall survival; MTD, maximum tolerated dose; mTTP, median time to progression; mDOR, median duration of response; NR, not reached; NA, not available; IV, intravenous; SC, subcutaneous.

### Venetoclax Single Agent

The phase 1 trial NCT01794520 evaluated the safety of venetoclax monotherapy in 66 patients with RRMM (33). Thirty patients were enrolled in the dose escalation part of the trial, while 36 patients were enrolled in the safety expansion phase. The patients received a median of 5 prior therapies (range: 1–15); approximately 60% of patients were bortezomib and lenalidomide double refractory. Thirty (46%) patients were positive for t(11;14). In terms of response, the ORR was 21% (14/66), and 15% achieved ≥VGPR. Most responses (12/14, 86%) were reported in patients with t(11;14). In this group, ORR was 40%, with 27% of patients achieving ≥VGPR. The MTD was not reached (NR), and the dose of 1200 mg/day was selected for the expansion cohort.

A real-world experience of 18 RRMM patients with t(11;14) at diagnosis treated with venetoclax as a single agent (starting with a dose of 100 mg daily and increasing to a maximum dose of 400 mg daily) was recently reported (44). Six patients (33%) achieved a response ≥PR; the dominant nonhematological adverse event (AE) was nausea, while the hematological AEs were neutropenia and thrombocytopenia.

### Venetoclax Plus Dexamethasone

The safety and efficacy of venetoclax was also evaluated in combination with dexamethasone in 51 RRMM patients with t(11;14) in an open-label phase 1/2 study (NCT01794520) (34). The phase 1/2 patients had respectively received a median of 3/5 lines of prior therapy, and 20/87% were refractory to daratumumab. At a median follow-up of 12.3/9.2 months, ORR was 60/48%. The DOR, estimated at 12 months, was 50/61%, and the median time to progression (mTTP) was 12.4/10.8 months.

### Venetoclax in Combination With Other Drugs

A single-center, retrospective study reported data on 47 patients with RRMM treated with off-label venetoclax (45) after a median of 7 (range: 3–13) lines of therapy; prior treatments also included autologous stem cell transplant (ASCT) in 39 patients (83%). Most patients (87%) received venetoclax plus a PI, though there was heterogeneity in the venetoclax-containing regimens. Eighteen patients (38%) were positive for t(11;14). The ORR was 39%, with 17% achieving ≥VGPR. In the t(11;14) group, ORR was 71%, with 24% achieving ≥VGPR. OS was 15.6 months, and mPFS was 2.1 months.

Venetoclax has been evaluated in combination with bortezomib and dexamethasone in 66 RRMM patients enrolled in a phase 1b study (NCT01794507) (35). In the dose escalation part of the study, 54 patients received venetoclax orally from 100 to 1,200 mg/day until progression after a 1-week lead-in period. In the safety expansion phase, 12 patients received venetoclax 800 mg daily until progression. The median number of prior lines of treatment was three. Nine patients (14%) were positive for t(11;14). Thirty-nine percent of the participants were refractory to bortezomib, and 53% were refractory to lenalidomide. Approximately 60% previously underwent ASCT. In terms of efficacy, ORR was 67%, including 20% CR/sCR and 23% VGPR. In the subgroup of patients that were not refractory to bortezomib and who had one to three prior therapies, ORR of 97% and ≥VGPR of 73% were observed.

In the randomized, double-blind, multicenter phase 3 trial BELLINI (NCT02755597), 291 patients with RRMM who had received one to three previous therapies were enrolled to receive venetoclax (194 patients) or placebo (97 patients) with bortezomib and dexamethasone (36). Treatment was given in 21-day cycles for the first eight cycles and 35-day cycles from the ninth cycle until PD, unacceptable toxicity, or patient withdrawal. Randomization was stratified by previous exposure to a PI and the number of previous therapies. ORR was 82% (venetoclax arm) *versus* 68% (placebo arm), and ≥VGPR was seen in 59 *versus* 36% of patients, respectively. In patients with t(11;14), ORR was 90% (venetoclax group) *versus* 47% (placebo group). mDOR was NR with venetoclax compared with 12.8 months with placebo. At the last update (37), with a median follow-up of 28.6 months, mPFS was 23.2 months with venetoclax *versus* 11.4 months with placebo; mOS was 33.5 months in the venetoclax group, while it was NR with the placebo group. There was an increased mortality in the venetoclax group (14 treatment-emergent deaths *versus* one in the placebo arm) mainly due to a higher rate of infection; as a consequence, in March 2019, FDA suspended the enrollment of new patients in this trial.

Venetoclax (800 mg/day), in combination with a standard dose of bortezomib and dexamethasone, was administered until PD or unacceptable toxicity in a real-life experience recently reported (46). Eleven patients with RRMM and highly pretreated with a median of 7 (range: 4–10) previous lines of therapy were included; all patients were negative for t(11;14). ORR was 27% (3/11), with one (9%) patient reaching VGPR and two (18%) patients reaching PR; two (18%) patients had a stable disease (SD), and six (54%) patients had PD. The mPFS of the whole cohort was 2 months. Nevertheless, the mPFS of those who responded with PR or better was 9 months *versus* 1.5 months for non-responders. The mOS of the whole cohort was 12 months (NR for PR or better *versus* 5 months for non-responders). The main AEs included gastrointestinal toxicities, especially nausea, thrombocytopenia, and infections.

In a phase 2 ongoing trial (NCT02899052), 43 patients with RRMM and no prior carfilzomib exposure were enrolled to receive venetoclax in combination with carfilzomib and dexamethasone (38, 39). The treatment continued until PD or unacceptable toxicity. Eight patients (19%) were positive for t(11;14). The median number of prior lines of therapy was 2 (range: 1–3). ORR was 79%, ≥CR rate was 40%, and ≥VGPR rate was 64% for all patients.

A real-world experience of 14 RRMM patients treated with venetoclax, carfilzomib, and dexamethasone was recently reported (47). The median previous number of therapies was 5 (range: 2–9). Five patients were positive for t(11;14). Regarding efficacy, ORR among all patients was 35,7%, with all responding patients in VGPR or better. Strikingly, these five responders specifically corresponded to the five t(11;14)-positive patients, resulting in 100% ORR for this particular cytogenetic subgroup and contrasting with the absence of response ≥PR in t(11;14)-negative patients. A rapid but short-lived response was reported in two further cases of patients with RRMM carrying t(11,14) and treated with venetoclax, carfilzomib, and dexamethasone (48).

At the 2021 EHA congress, real-world data of 50 MM patients with t(11;14) have been reported; most patients received venetoclax in combination with a PI and dexamethasone (49). The ORR was remarkably high (48/50 patients responded to the treatment with CR of 28%, VGPR of 38%, and PR of 30%), given that 33 patients (66%) of this group were heavily pretreated. The calculated PFS and OS were 15.5 and 24 months, respectively. The most common AEs were cytopenia, gastrointestinal toxicities, and infections.

Notably, a phase 1/2 study (NCT03399539) aiming to determine the MTD of venetoclax in combination with ixazomib and dexamethasone (phase 1) and to evaluate the therapeutic activity of this triplet in patients with RRMM (phase 2) has been temporarily closed (by FDA and IRB) to enrollment due to safety-related findings (41).

Regarding the combination of venetoclax plus pomalidomide, in the ongoing multicenter, randomized, open-label phase 3 study CANOVA (NCT03539744), RRMM patients with t(11;14) will be randomized 1:1 to venetoclax or pomalidomide plus dexamethasone (40). The treatment will continue until PD, unacceptable toxicity, or withdrawal from the study. The patients will be stratified at screening and before randomization according to age, prior lines of therapy, and International Staging System stage. Furthermore, in another phase 2 trial (NCT03567616), venetoclax will be combined with pomalidomide and dexamethasone in RRMM patients with at least one prior line of therapy (41). The study will include a dose escalation phase and a dose expansion phase, where the participants will be divided into two cohorts based on the presence of t(11;14).

Some studies are exploring the role of venetoclax in combination with MoAbs. An ongoing phase 1/2, non-randomized, multicenter study (NCT03314181) is evaluating the safety, efficacy, and pharmacokinetics of venetoclax, daratumumab, and dexamethasone (VenDd) +/- bortezomib (V) in RRMM (42). The study consists of three distinct parts: part 1 and 2 include patients with t(11;14) or irrespective of t(11;14), respectively, who receive VenDd; part 3 enrolls patients with t(11;14) who receive VenDd +/- bortezomib. The median follow-up time (VenDd/VenDVd) was 10 and 9 months. The ORR in VenDd/VenDVd was 96/92%, and 96/79% had ≥VGPR rate. The mPFS and mDOR were not reached.

An open-label, randomized, multicenter, three-arm phase 1b/2 study (NCT03312530) of cobimetinib (a MEK inhibitor) administered as a single agent and in combination with venetoclax +/- atezolizumab (an engineered MoAb of IgG1 isotype against protein programmed cell death-ligand 1) is currently under investigation in 49 RRMM patients who had received three to five prior therapies, including a PI and an IMiD (43). The patients are randomized 1:2:2 to cobimetinib (arm A), cobimetinib+venetoclax (arm B), or cobimetinib+venetoclax+atezolizumab (arm C). The median prior line of therapy was 4 (range: 3–5), with prior ASCT in 43% and prior daratumumab in 41% of patients, respectively. Twenty-four percent of the patients had high-risk cytogenetics. The ORR was 0% (arm A), 27% (arm b), and 29% (arm C), while the mOS in the three arms were 12.9, 12.4, and 23.3 months, respectively.

Finally, various case reports have been published about the use of venetoclax monotherapy or in combination with other drugs in patients with advanced RRMM, particularly in patients with primary or secondary plasma cell leukemia (50–60).

To date, there is no data (or active clinical trials) evaluating the role of venetoclax in NDMM; there is no data as well on any potential impact on stem cells and stem cell collection. A trial (NCT03785184) aimed to evaluate the safety and preliminary efficacy of venetoclax when combined with lenalidomide and dexamethasone in patients with NDMM and positive for t(11;14), first available on *ClinicalTrials.gov* in December 2018, was withdrawn (41).

## Selinexor

Selinexor is a first-in-class, oral, slowly reversible, highly specific inhibitor of exportin-1 (XPO-1) which is an important nuclear exporter for more than 200 proteins, including many tumor-suppressor proteins (TSPs). The overexpression of XPO-1 in myeloma cells, as in most cancer cells, makes selinexor a promising targeted therapy (61) for MM patients. It prevents the transport of TSPs from the nucleus to the cytoplasm, leading to the accumulation of TSPs in the nucleus with consequent cell cycle arrest and apoptosis of MM cells (**Figure 1**) (62, 63), without affecting the normal cells (64). The anticancer activity of XPO-1 inhibitors (including selinexor) is p53 mutation independent (65) and is synergistically increased when combined with other chemotherapies and targeted therapies (66–69); the combination with glucocorticoids would intensify the anti-myeloma activity, too (70). Moreover, selinexor, inhibiting NF-kB, seems to reduce in the microenvironment of cytokines which are vital for the survival of MM cells, like IL-6, IL-10, and VEGF (65). Selinexor has recently been approved by the US FDA in combination with dexamethasone for RRMM patients who have received at least four prior therapies and whose disease is refractory to at least two PIs, at least two IMiDs, and an anti-CD38 mAb (71). A detailed summary of the main clinical trials on selinexor monotherapy or in combination in the setting of RRMM, including schedules and doses, can be found in **Table 3**.

**Table 3:** Summary of findings of main clinical trials with **Selinexor **in relapsed/refractory multiple myeloma.

Regimen (trial ID)	Phase/number of patients	Dosing	Median number of prior lines(range)	Efficacy	Adverse events (grade 3 and 4)	Reference
Selinexor +/- Dexamethasone (**NCT01607892**)	I/84	**Dose escalation phase** (25 patients: MM (22) or/and Waldenstrom macroglobulinemia (3): **Selinexor **(3–60 mg/m^2^) in eight or 10 doses per 28-day cycle. **Dose expansion phase** (59 MM patients): **Selinexor **(45 or 60 mg/m^2^) plus **Dexamethasone** (20 mg), twice weekly in 28-day cycles, or **Selinexor **(40 or 60 mg flat dose) without corticosteroids in 21-day cycles	6 (1–16)	ORR: 10% mDOR: 5 months (2–11)	Thrombocytopenia (45%), anemia (23%), neutropenia (23%)	Chen C et al. (63)
Selinexor plus Dexamethasone (STORM, **NCT02336815**)	IIb/201	**Part 1** (79 patients): (A) **Selinexor **(80 mg), **Dexamethasone** (20 mg) twice weekly on days 1 and 3 for 3 weeks of each 4-week cycle (B) **Selinexor **(80 mg), **Dexamethasone** (20 mg) twice weekly continuously in 4-week cycles **Part 2** (122 patients): **Selinexor **(80 mg), **Dexamethasone** (20 mg) twice weekly on days 1 and 3, until disease progression	7 (3–17) 7 (3–18)	ORR: 21% mDOR was 5 months mPFS: 2.3 months mOS: 9.3 months ORR: 26% mDOR: 4.4 months mPFS: 3.7 months mOS: 8.6	Thrombocytopenia (59%), anemia (28%), neutropenia (23%), hyponatremia (22%), leukopenia (15%), and fatigue (15%) Thrombocytopenia (59%), anemia (44%), hyponatremia (22%), neutropenia (21%), nausea (10%)	Vogl DT et al. (70) Chari A et al. (67)
Selinexor pus Dexamethasone (MARCH, **NCT03944057**)	II/60	**Selinexor **(80 mg twice weekly of each 28-day cycle), **Dexamethasone** (20 mg twice weekly of each 28-day cycle)	5 (1–16)	ORR: 26.7% mDOR: 4.6 months mPFS: 3.7 months mOS: NR	anemia (60%), thrombocytopenia (55%), leukopenia (42%), lymphopenia (42%), neutropenia (38%), hyponatremia (28%), and pneumonia (23%)	Fu W et al. (72)
Selinexor plus Pomalidomide and Dexamethasone (STOMP, **NCT02343042**)	Ib/II 65	**Selinexor **(once weekly: 60, 80, or 100; twice weekly: 60 or 80 mg), **Pomalidomide **(2, 3, or 4 mg) on days 1–21 of each 28-day cycle; **Dexamethasone** (20 mg twice weekly or 40 mg once weekly) **RP2D: Selinexor **(60 mg once weekly), **Pomalidomide **(4 mg) on days 1–21 of each 28-day cycle, **Dexamethasone** (40 mg once weekly)	3 (1–10)	**Pomalidomide-naïve** (44 patients) ORR: 57% mPFS: 12.2 months **Pomalidomide-exposed** (16 patients) ORR:44%	Neutropenia (55%), anemia (32%), thrombocytopenia (31%), fatigue (11%), decreased appetite (2%), nausea (2%)	White DJ et al. (73)
Selinexor plus Lenalidomide and Dexamethasone (STOMP, **NCT02343042**)	Ib/II 24	**Selinexor **(once weekly: starting dose 80 mg; twice weekly: starting dose 60 mg), **Lenalidomide **(25 mg) on days 1–21 of each 28-day cycle, **Dexamethasone** (40 mg once weekly or 20 mg twice weekly) **RP2D: Selinexor **(60 mg once weekly), **Lenalidomide **(25 mg) on days 1–21 of each 28-day cycle, **Dexamethasone** (40 mg once weekly)	1.5 (1–8)	**Lenalidomide-naïve** (12 patients) ORR: 92% PFS: NR **Lenalidomide-exposed** (eight patients) ORR: 13%	Thrombocytopenia (63%), neutropenia (63%), nausea (4%), fatigue (17%), decreased appetite (8%), weight loss (8%)	White DJ et al. (74)
Selinexor plus Daratumumab and Dexamethasone (STOMP, **NCT02343042**)	Ib/II 34	**Selinexor **(once weekly: 100 mg; twice weekly: 60 mg) in 28‐day cycles; **Daratumumab **(16 mg/kg weekly for weeks 1–8, every 2 weeks for weeks 9–24, then every 4 weeks for weeks ≥25); **Dexamethasone** (40 mg once weekly) **RP2D: Selinexor **(100 mg once weekly), **Daratumumab **(16 mg/kg weekly for weeks 1–8, every 2 weeks for weeks 9–24, then every 4 weeks for weeks ≥25), **Dexamethasone** (40 mg once weekly)	3 (2–10)	**Daratumumab-naïve** (32 patients) ORR: 73% mPFS: 12.5 months	Thrombocytopenia (47.1%), anemia (32.4%), leukopenia (32.4%), neutropenia (26.5%), fatigue (17.6%), nausea (8.8%), hyponatremia (11.8%)	Gasparetto C et al. (75)
Selinexor plus Carfilzomib and Dexamethasone (STOMP, **NCT02343042**)	Ib/II 27	**Selinexor **(80 or 100 mg once weekly), **Carfilzomib** (56 or 70 mg/m^2^) on days 1, 8, and 15 of 28-day cycle; **Dexamethasone** (40 mg) once weekly **RP2D: Selinexor **(80 mg once weekly), **Carfilzomib** (56 mg/m^2^) on days 1, 8, and 15 of 28-day cycle; **Dexamethasone** (40 mg) once weekly	4 (1–8)	ORR: 78% mPFS: 23.7 months	Thrombocytopenia (56%), anemia (19%), neutropenia (7%), fatigue (7%), anorexia (4%)	Gasparetto C et al. (76)
Selinexor plus Bortezomib and Dexamethasone (STOMP, **NCT02343042**)	Ib/II 42	**Cohort 1. Selinexor **(80 or 100 mg once weekly in a 35-day cycle), **Dexamethasone** (40 mg) once weekly, **Bortezomib **(1.3/m^2^) on days 1, 8, 15, and 22 **Cohort 1. Selinexor **(80 mg once weekly in a 21-day cycle), **Dexamethasone** (40 mg) once weekly, **Bortezomib **(1.3/m^2^) on days 1, 4, 8, and 11 **Cohort 2. Selinexor **(60 or 80 mg twice weekly in a 35-day cycle); **Dexamethasone** (20 mg) on days 1, 3, 8, 10, 15, 17, 22, 24, 29, and 31; **Bortezomib **(1.3/m^2^) on days 1, 8, 15, and 22 **RP2D: Selinexor **(100 mg once weekly), **Bortezomib **(1.3 mg/m^2^) once weekly for 4 weeks, **Dexamethasone** (40 mg) once weekly per 35-day cycle	3 (1–11)	**Global ORR: 63%** ORR PI non-refractory: 84% ORR PI refractory: 43% **Global mPFS: 9.0 months** mpfs PI non-refractory: 17.8 months, mPFS PI refractory: 6.1 months	Thrombocytopenia (45%), neutropenia (24%), fatigue (14%), anemia (12%)	Bahlis NJ et al. (77)
Selinexor plus Ixazomib and Dexamethasone (**NCT02831686**)	I/18	**Selinexor** Cohort A: 40 and 60 mg on days 1, 3, 8, 10, 15, and 17 of a 28-day cycle Cohort B: 80 and 100 mg on days 1, 8, 15, and 22 of each 28- day cycle **Ixazomib **(4 mg) on days 1, 8, and 15 of each 28-day cycle **Dexamethasone**: the same days as selinexor	5 (1–11)	ORR: 22%, maximum DOR: 14 months	Thrombocytopenia (61%), neutropenia (28%), anemia (17%), nausea (11%), vomiting (11%), fatigue (11%)	Salcedo M et al. (78)
Selinexor plus Carfilzomib and Dexamethasone (SINE, **NCT02199665**)	I/21	**Selinexor **(20, 30, 40, and 60 mg) on days 1, 3, 8, 10, 15, and 17 of a 28-day cycle; **Carfilzomib** (20, 20/27, 20/36, 20/45, and 20/56 mg/m^2^): cycle 1–8 on days 1 and 2, 8 and 9, 15 and 16; cycle 9+: on days 1 and 2, 15 and 16; **Dexamethasone**: 40 mg weekly (cycle 1–4), 20 mg weekly (cycle 5+) **RP2D: Selinexor** (60 mg) on days 1, 3, 8, 10, 15, and 17, **Carfilzomib** (20/27 mg/m^2^) on days 1, 2, 8, 9, 15, and 16; **Dexamethasone** (20 mg; 10 mg from cycle 5 afterwards) on days 1, 2, 8, 9, 15, 16, 22, and 23 on a 28-day cycle	4 (2–10)	ORR: 48% CBR: 71% mPFS: 3.7 months mOS: 22.4 months	Thrombocytopenia (71%), anemia (33%), neutropenia (33%), lymphopenia (33%), infections (24%)	Jakubowiak AJ et al. (79)
Selinexor plus Doxorubicin and Dexamethasone (**NCT02186834**)	I/27	Loading phase (1 to 2 weeks): A: **Selinexor**, **Dexamethasone** twice weekly for 2 weeks or B: one dose of **Selinexor **and **Dexamethasone** Induction phase: **Doxorubicin** (20 mg/m^2^ IV) on day 1, **Selinexor**, and **Dexamethasone** (once weekly) Maintenance phase: **Selinexor **and **Dexamethasone** (once weekly) **RP2D: Selinexor **(80 mg on days 1, 8, and 15), **Doxorubicin** (20 mg/m^2^ on day 1), and **Dexamethasone** (40 mg on days 1, 8, and 15)	6 (2–10)	ORR: 15% CBR: 26%	Thrombocytopenia 33%, neutropenia 33%, hyponatremia 30%, anemia 26%, nausea/vomiting 11%, hyperglycemia 11%, diarrhea 7%, fatigue 7%	Baz R et al. (80)
Selinexor, Bortezomib, and Dexamethasone (SVd) *vs*. Bortezomib and Dexamethasone (Vd) (BOSTON, **NCT03110562**)	III/402	**SVd (195 patients): Selinexor **(100 mg once weekly), **Bortezomib **(1–3 mg/m^2^ once weekly), **Dexamethasone** (20 mg twice weekly) **Vd (207 patients): Bortezomib **(1–3 mg/m^2^ twice weekly for the first 24 weeks and once weekly thereafter), **Dexamethasone** (20 mg four times per week for the first 24 weeks and twice weekly thereafter)	2 (1–3)	**SVd:** mPFS: 13.93 months ORR: 76.4% **Vd:** mPFS: 9.46 months ORR: 62.3%	**SVd:** thrombocytopenia: 39%, fatigue 13%, anemia 16%, pneumonia 11% **Vd:** thrombocytopenia: 17%, fatigue 1%, anemia 10%, pneumonia 11%	Grosicki S et al. (81)
Selinexor plus Bortezomib, Dexamethasone, Daratumumab (SELIBORDARA, **NCT03589222**)	II/ongoing	**Selinexor **(100 mg weekly out of each 4-week cycle), **Dexamethasone** (40 or 20) with each dose of selinexor, **Daratumumab **(16 mg/kg IV) on days 1, 8, 15, and 22 during the first two cycles; on days 1 and 15 during cycles 3 to 6 and on day 1 thereafter; **Bortezomib **(1.3 mg/m^2^) on days 1, 8, 15, and 22 starting from the first cycle and on days 1 and 15 since cycle 9. Each cycle is 4 weeks in duration	NA	NA	NA	https://clinicaltrials.gov/ (41)
Selinexor, Cyclophosphamide, Prednisolone *vs*. Cyclophosphamide and Prednisolone (MUKTWELVE, **ISRCTN15028850**)	II/ongoing	**SCP: Selinexor **(100 mg once a week) on days 1, 8, 15, and 22; **Cyclophosphamide** (oral 50 mg once daily, starting on day 1), **Prednisolone** (oral 30 mg every other day, starting on day 1) **CP: Cyclophosphamide** (oral 50 mg once daily, starting on day 1), **Prednisolone** (oral 30 mg every other day, starting on day 1), followed by SCP combination	NA	NA	NA	Brown SR et al. (82)

pts, patients; ORR, overall response rate; VGPR, very good partial response; PR, partial response; PD, progressive disease; CBR, clinical benefit rate; mPFS, median progression free-survival; mOS, median overall survival; mTTP, median time to progression; mDOR, median duration of response; NR, not reached; NA, not available; IV, intravenous.

The multicenter phase I clinical trial (NCT01607892) was conducted in advanced hematological malignancies to assess the safety, efficacy, and recommended phase 2 dose of selinexor. In the dose escalation phase, 22 patients with heavily pretreated MM and three with Waldenstrom macroglobulinemia were administered with selinexor as a single agent. In the dose expansion phase, 59 patients with MM received selinexor in combination with dexamethasone. Considering all patients, the ORR was 10%; considering patients treated with selinexor at 45 mg/m^2^ twice weekly plus dexamethasone, the ORR was 50% (63).

The single-arm, open-label, multicenter phase 2b study STORM (NCT02336815) evaluated selinexor plus dexamethasone in patients with MM previously treated with lenalidomide, pomalidomide, bortezomib, carfilzomib, and daratumumab and refractory to prior treatment with glucocorticoids, an IMiD, a PI, and daratumumab (70). This study consisted of two parts: part 1 included 79 patients with both quad-refractory MM and penta-refractory MM, and part 2 included 122 patients with penta-refractory MM only. Regarding part 1, the ORR was 21%, mDOR was 5 months, and mPFS and mOS were 2.3 and 9.3 months, respectively. Regarding part 2, the ORR was 26%, mDOR was 4.4 months, and mPFS and mOS were 3.7 and 8.6 months, respectively (67).

The MAMMOTH study evaluated the efficacy of selinexor and dexamethasone in a cohort of patients similar to those enrolled in the STORM study *versus* other multi‐agent combinations in RRMM patients treated in academic centers after they became refractory to anti‐CD38 mAbs (including a subset of patients who were triple‐class refractory) (83). In this retrospective analysis, selinexor plus dexamethasone improved OS (10.4 *versus* 6.9 months) and ORR (32.8 *versus* 25%) with respect to contemporary care (without selinexor).

The single-arm phase 2 MARCH study (NCT03944057) evaluated selinexor and dexamethasone in RRMM patients in China. At the last update (72), 60 patients have been enrolled; the ORR was 26.7%, mDOR was 4.6 months, mPFS was 3.7 months, mOS was NR, and the OS rate at 9 months was 68.5%.

STOMP (NCT02343042) is a phase Ib/II multicenter, open-label, clinical trial with the goals of determining the MTD, the recommended phase 2 dose (RP2D), and the efficacy and safety of selinexor and dexamethasone in combination with various widely used anti-myeloma drugs (bortezomib, pomalidomide, lenalidomide, carfilzomib, daratumumab, *etc.*) in patients with RRMM or NDMM.

Sixty-five RRMM patients were enrolled in the STOMP trial (NCT02343042) to receive selinexor, dexamethasone, and pomalidomide after a median of 3 (range: 1–10) prior therapies (73). The RP2D was selinexor 60 mg, pomalidomide 4 mg, and dexamethasone 40 mg. Among pomalidomide-naïve patients (*n* = 44), the ORR was 57% (1 sCR, 1 CR, 8 VGPRs, and 15 PRs), and mPFS was 12.2 months. In patients treated with RP2D (*n* = 20), the ORR was 65% (1 sCR, 5 VGPRs, and 7 PRs); mPFS was NR, with a median follow-up time of 3.9 months. In pomalidomide-refractory patients (*n* = 16) and those with prior exposure to daratumumab (*N* = 15), the ORR was 44 and 60%, respectively.

Twenty-four RRMM patients were enrolled in the STOMP trial (NCT02343042) to receive selinexor, dexamethasone, and lenalidomide (74). The median number of prior treatments was 1.5 (range: 1–8). RP2D was set at 60 mg of selinexor, dexamethasone 40 mg, and lenalidomide 25 mg. Regarding outcome, among the lenalidomide-naïve patients (*n* = 12), the ORR was 92%, including one sCR, four VGPR, and six PR. PFS has not been reached, with a median follow-up period of 7.8 months. For patients with prior lenalidomide treatment (*n* = 8), the ORR was 13%, suggesting that selinexor–lenalidomide–dexamethasone is effective for patients with RRMM who have not been previously exposed to lenalidomide.

Selinexor, in combination with daratumumab and dexamethasone, has been evaluated, within the STOMP trial (NCT02343042), in 34 RRMM patients who had received three or more prior lines of therapy, including a PI and an IMiD, or whose MM was refractory to a PI and an IMiD (75). The median number of prior therapies was 3 (range: 2–10). The RP2D was selinexor 100 mg weekly, daratumumab 16 mg/kg (weekly for weeks 1–8, every 2 weeks for weeks 9–24, and then every 4 weeks for weeks ≥25), and dexamethasone 40 mg weekly. The ORR was 73%, and mPFS was 12.5 months in daratumumab-naïve patients (*n* = 32).

Twenty-seven RRMM patients were enrolled in the STOMP trial (NCT02343042) to receive selinexor, carfilzomib, and dexamethasone (76). The median number of prior regimens was 4 (range: 1–8). The RP2D was selinexor 80 mg, carfilzomib 56 mg/m^2^, and dexamethasone 40 mg. The ORR was 78% (5 CRs, 8 VGPRs, and 8 PRs), and mPFS was 23.7 months.

Another study evaluating the efficacy of selinexor in combination with carfilzomib and dexamethasone is the phase 1 SINE trial (NCT02199665). Twenty-one RRMM patients had been enrolled after a median of four prior lines of therapy, whereas 95% had received carfilzomib and 81% were dual-class refractory (PI and IMiD) and previously exposed to bortezomib, carfilzomib, lenalidomide, and pomalidomide (79). The RP2D was set at 60 mg of selinexor, carfilzomib at 20/27 mg/m^2^, and dexamethasone at 20 mg. The ORR was 48%, CBR was 71%, and mPFS and mOS for all enrolled patients were 3.7 and 22.4 months, respectively.

Returning to the STOMP trial (NCT02343042), 42 patients with RRMM were enrolled to receive selinexor, dexamethasone, and bortezomib (77). The median number of prior lines of therapy was 3 (range: 1–11). Fifty percent of the patients were refractory to a prior PI (bortezomib, carfilzomib, or ixazomib), and 45% were refractory to both a PI and an IMiD (lenalidomide, pomalidomide, or thalidomide). The RP2D was set as selinexor at 100 mg, bortezomib at 1.3 mg/m^2^, and dexamethasone at 40 mg. The ORR for the entire population was 63%: 84% ORR for PI non-refractory and 43% for PI-refractory patients. The mPFS for all patients was 9.0 months; 17.8 months for PI non-refractory and 6.1 months for PI-refractory patients.

In the open-label phase 3 trial BOSTON (NCT03110562), 402 RRMM patients were randomly allocated to receive bortezomib, dexamethasone (Vd) +/- selinexor (S) (SVd: 195 patients; Vd: 207 patients) (81). Randomization was done using interactive response technology and stratified by previous PI exposure, lines of treatment, and MM stage. Crossover to SVd upon progression on Vd was allowed. The median number of prior lines of therapy was 2 (range: 1–3). After a median follow-up period of 13.2 months for SVd and 16.5 months for Vd, mPFS was significantly longer in the SVd group (13.93 months) than in the Vd group (9.46 months). The ORR in the SVd group was 76.4% (*versus* 62.3% of the Vd group) and included 19 sCR, 14 CR, 54 VGPR, and 62 PR. mDOR was longer with SVd (20.3 months) than with Vd (12.9 months). Furthermore, the median time to next anti-MM treatment was longer in the SVd group (16.1 months) than in the Vd group (10.8 months). Efficacy was consistent across various patient subgroups, including patients with high-risk cytogenetic abnormalities. At the 2021 ASCO congress, a *post-hoc* analysis of this study comparing the survival benefits in patients ≥65 *versus <*65 years of age was reported; for patients ≥65 years, mOS was NR with SVd, while it was 28.6 months with Vd; for patients <65 years, there was no difference in terms of OS (84). Another *post-hoc* analysis (85) reported an improved ORR, PFS, and time-to-next-treatment in the SVd group *versus* Vd regardless of the documented refractory status to lenalidomide or any IMiDs.

In a real-life experience report, eight RRMM, heavily treated patients and with a median of 11 prior lines of therapy (range: 6–18), received a treatment based on the dosing schedule of SVd of the BOSTON trial (86). The responses included one CR, one VGPR, two PR, three SD, and one PD. The mPFS was 91 days (range: 58–350), while OS was 300 days (range: 68–376). The treatment-related adverse effects (TRAEs) included fatigue, thrombocytopenia, and neutropenia, which were managed with selinexor dose adjustment and supportive care.

Another real-world experience included 13 RRMM patients, heavily treated and with a median of 7 (range: 4–10) prior lines of therapy; the patients received selinexor (40–80 mg), dexamethasone (20–40 mg), and bortezomib (1.3 mg/m^2^) once a week (87). The ORR was 23% (the responses included three VGPR, one MR, five SD, and four PD). The adverse events were in line with the known safety profile of each of the components.

Selinexor was administered in combination with ixazomib and dexamethasone to 18 heavily pretreated MM patients in a phase I, open-label trial (NCT02831686) (78). Cohort A had a bi-weekly dosing of selinexor with two dose levels (40 and 60 mg). Cohort B had a weekly dosing of selinexor with two dose levels (80 and 100mg). The patients had a median of five prior lines of therapy, and 83% were PI refractory. The ORR was 22%, and the maximum DOR was 14 months. The once-weekly schedule was preferred due to better tolerability, and the selinexor MTD was determined at 80 mg.

In a multicenter, open-label phase I/II clinical trial (NCT02186834), selinexor was administered in combination with doxorubicin and dexamethasone in 27 RRMM patients (80). The median number of prior regimens was 6 (range: 2–10). The RP2D was selinexor (80 mg), doxorubicin (20 mg/m^2^), and dexamethasone (40 mg). The ORR was 15%, and CBR was 26%.

The ongoing open-label, multicenter phase II trial, SELIBORDARA (NCT03589222), aims to evaluate the efficacy and safety of the combination of selinexor, bortezomib, dexamethasone, and daratumumab in RRMM patients (41).

The ongoing randomized, controlled, open, parallel group, multi-center phase II trial, MUKTWELVE (ISRCTN15028850), aims instead to evaluate the clinical efficacy of selinexor in combination with cyclophosphamide and prednisolone in patients with RRMM (82). A maximum of 60 participants will be recruited.

Among other selinexor trials with available results, seven patients received a selinexor-based regimen (one selinexor–dexamethasone, one selinexor–bortezomib–dexamethasone, and five selinexor-carfilzomib-dexamethasone) after progression on CAR T cell therapy (88). All of them were heavily pretreated, with a median of 10 prior lines of treatment; four were penta-refractory and had a rapidly progressive disease. The responses to selinexor-based regimens were one sCR, three VGPR, two PR, and one minimal response. Although preliminary, these data suggest the effectiveness of the selinexor-based regimen also after CAR T cell therapy.

Regarding the role of selinexor in the treatment of NDMM, limited data are available as well as data on any potential impact on stem cell collection. In the STOMP trial (NCT02343042), eight NDMM patients were enrolled to receive the RP2D of selinexor (60 mg once weekly), lenalidomide (25 mg, on days 1–21 of each 28-day cycle), and dexamethasone (40 mg once weekly) (74). All seven patients evaluable for efficacy achieved a response, with an ORR of 100%, including 1 CR, 4 VGPR, and 2 PR. With a median follow-up of 10.2 months, the median PFS has not been reached. The common TRAEs grade ≥3 were thrombocytopenia (38%), neutropenia (75%), fatigue (50%), and decreased appetite (13%). Out of these seven patients, three withdrew their consent to transit to successful autologous stem cell collection and transplantation.

Twelve patients were enrolled in phase I/II of NCT02780609 to receive selinexor (dose level 1: 40 mg, dose level 2: 60 mg, and dose level 3:80 mg) on days -3 and -2 before melphalan, in combination with high-dose melphalan (100 mg/m^2^ IV on days -3 and -2), as a conditioning regimen for hematopoietic cell transplant (89). The primary objective was to establish the MTD and identify the RP2D. The combination with selinexor 80 mg (RP2D) with high-dose melphalan at 100 mg/m^2^ on days -3 and -2 was well tolerated, and the engraftment kinetics were not altered (neutrophil engraftment occurred with a median of 11 days, and platelet engraftment occurred with a median of 15 days). The trial is proceeding to phase II to assess the efficacy of this combination.

SeaLAND (ALLG MM23) is an ongoing randomized phase 3 trial regarding maintenance after ASCT in NDMM. It aims to compare standard lenalidomide maintenance after ASCT with a low dose of selinexor and lenalidomide to find any benefits in terms of CR, minimal residual disease negativity rate, and PFS (90).

Considering the promising results of selinexor, a second-generation oral selective inhibitor of nuclear export, eltanexor (KPT-8602), is being evaluated in RRMM patients for safety and tolerability; 36 patients were enrolled in a phase I/II open-label study NCT02649790 (91). Based on preliminary data, eltanexor has been shown to have a potentially improved adverse effect profile with similar efficacy compared with selinexor, although more clinical data are needed at this time.

## Conclusion

Recent therapeutic regimens based on melflufen, venetoclax, or selinexor provide a promising novel approach to patients with RRMM, even outside of the strict immunotherapy treatments. In particular, melflufen, in combination with dexamethasone alone or with a third agent, has shown effectiveness in triple-class refractory patients and in extramedullary disease that represent a major issue in the context of aggressive MM progression (92). Venetoclax appears to be particularly effective in patients with t (11,14), which is present in approximately 20% of MM (93). Selinexor also shows promising outcomes in terms of ORR; the responses observed in selinexor-based three-drug regimens are higher as compared to two-drug regimens, providing a benchmark for further studies (94). Regarding the side effects, TRAEs are generally reversible by applying dose modification and appropriate supportive care (95) to reduce their incidence and maximize the effectiveness of therapy. However, there have been treatment-emergent AEs associated with agents such as venetoclax and Selinexor, and therefore, in some circumstances, the risk–benefit profile may not be favorable compared to currently approved regimens. Obviously, patient selection is necessary for determining the optimal combination of melflufen, venetoclax, and selinexor with other approved agents according to MM biology and status, previous drugs, disease biomarkers, and patient clinical features. Well-designed, pivotal clinical trials are needed to further investigate these agents, preferably in combination and possibly in earlier lines of treatment where these agents could provide a higher benefit. If so, the exact position of these drugs in the therapeutic path of patients with MM will become evident. Currently, potent next-generation cereblon E3 ligase modulators (CELMods), such as iberdomide and CC-92480, not strictly considered as immunotherapy approaches, are in clinical development (96). Though outside of the scope of our review, these new agents have the potential to replace backbone IMiDs and PIs and should also be considered within the expanding number of active agents as a further opportunity and challenge to combine and sequence therapies to maximize long-term patient survival and quality of life.

## Author Contributions

NS and PM analyzed the data and conceived and wrote the paper. PC and RR reviewed pertinent literature and provided criticisms and suggestions. All authors contributed to the article and approved the submitted version.

## Conflict of Interest

The authors declare that the research was conducted in the absence of any commercial or financial relationships that could be construed as a potential conflict of interest.

## Publisher’s Note

All claims expressed in this article are solely those of the authors and do not necessarily represent those of their affiliated organizations, or those of the publisher, the editors and the reviewers. Any product that may be evaluated in this article, or claim that may be made by its manufacturer, is not guaranteed or endorsed by the publisher.

## Abbreviations

MM, multiple myeloma; RRMM, relapsed/refractory multiple myeloma; NDMM, newly diagnosed multiple myeloma; PIs, proteasome inhibitors; IMiDs, immunomodulatory agents; mAb, monoclonal antibodies; ORR, overall response rate; sCR, stringent complete response; CR, complete response; VGPR, very good partial response; PR, partial response; SD, stable disease; PD, progressive disease; CBR, clinical benefit rate; mOS, median overall survival; mPFS, median progression free-survival; mDOR, median duration of response; MTD, maximum tolerated dose; RP2D, recommended phase 2 dose; TRAEs, treatment-related adverse events; AE, adverse event; NR, not reached; ASCT, autologous stem cell transplant.
